# Association of Combined *p73* and *p53* Genetic Variants with Tumor HPV16-Positive Oropharyngeal Cancer

**DOI:** 10.1371/journal.pone.0035522

**Published:** 2012-04-16

**Authors:** Zhongqiu Wang, Erich M. Sturgis, Wei Guo, Xicheng Song, Fenghua Zhang, Li Xu, Qingyi Wei, Guojun Li

**Affiliations:** 1 Department of Head and Neck Surgery, The University of Texas MD Anderson Cancer Center, Houston, Texas, United States of America; 2 Department of Radiology, East Hospital, Tongji University School of Medicine, Shanghai, China; 3 Department of Epidemiology, The University of Texas MD Anderson Cancer Center, Houston, Texas, United States of America; 4 Department of Otolaryngology-Head and Neck Surgery, Beijing Tongren Hospital, Capital Medical University, Beijing, China; 5 Department of Otolaryngology-Head and Neck Surgery, Yuhuangding Hospital of Qingdao University, Yantai, China; 6 Department of General Surgery, Hebei General Hospital, Shijiazhuang, China; IPO, Inst Port Oncology, Portugal

## Abstract

p53 and p73 interact with human papillomavirus (HPV) E6 and E7 oncoproteins. The interplay between p53 and p73 and HPV16 may lead to deregulation of cell cycle and apoptosis, through which inflammation/immune responses control the HPV clearance and escape of immune surveillance, and subsequently contribute to tumor HPV16 status. In this case-case comparison study, HPV16 status in tumor specimens was analyzed and *p53* codon 72 and *p73* G4C14-to-A4T14 polymorphisms were genotyped using genomic DNA from blood of 309 oropharyngeal cancer patients. Odds ratios (ORs) and 95% confidence intervals (95% CIs) were calculated in univariate and multivariable logistic regression models to examine the association. The results from this study showed both *p53* variant genotypes (Arg/Pro+Pro/Pro) and *p73* variant genotypes (GC/AT+AT/AT) were significantly associated with HPV16-positive tumor in oropharyngeal cancer patients (OR, 1.9, 95% CI, 1.1–3.3 and OR, 2.1, 95% CI, 1.2–3.8, respectively), while the combined variant genotypes (*p53* Pro carriers and *p73* AT carriers) exhibited a significantly greater association with HPV16-positive tumor (OR, 3.2, 95% CI, 1.4–7.4), compared with combined wild-type genotypes (*p53* Arg/Arg and *p73* GC/GC), and the association was in a statistically significant dose-effect relationship (*p* = 0.001). Moreover, such association was more pronounced among several subgroups. These findings suggest that variant genotypes of *p53* and *p73* genes may be individually, or more likely jointly, associated with tumor HPV16-positive oropharyngeal cancer patients, particularly in never smokers. Identification of such susceptible biomarkers would greatly influence on individualized treatment for an improved prognosis.

## Introduction

Squamous cell carcinoma of the head and neck (SCCHN) typically presents in advanced stages and is associated with poor survival and high recurrence and second primary tumor rates [1]. Tobacco smoking and alcohol drinking are still the primary risk factors for SCCHN [2], but the incidence of oropharyngeal cancer is increasing especially in patients who are not smokers and alcohol abusers [3], [4], attributed mainly to the human papillomavirus (HPV). The absolute survival rates with chemoradiotherapy, a popular treatment approach for oropharyngeal cancers, have remained modest [5], whereas advanced oropharyngeal cancers appear to benefit from minimally invasive surgical approaches plus adjuvant therapy [6]. Several studies have compared the survival between HPV-negative patients and HPV-positive patients (chiefly oropharynx patients) [7]–[11], but the impact of HPV-positivity on survival is inconsistent. Therefore, further studies are needed to understand susceptibility for and modifying factors of the HPV16 carcinogenic process, which will facilitate individualized treatment for oropharyngeal cancers.

The prognosis for oropharyngeal cancer patients is in part explained by current staging and imaging techniques, while an identification of HPV associated oropharyngeal cancer may have important prognostic implications. Although HPV tumor positivity confers a favorable outcome, independent of other significant confounding factors including stage, treatment, smoking, etc, HPV-positive cancers are more likely to have a later stage, nodal involvement and advanced grade compared to HPV-negative cancers [12]. These facts may promote consideration for a new staging system for oropharyngeal cancer, stratified by HPV status. Thus, identification of new biomarkers for tumor HPV status detection (e.g., HPV DNA in tumors) may help ensure appropriate therapy for a better clinical outcome.

Both p53 and p73 can be activated by oncogenic signals, such as those derived from HPV DNA genome integration in the nucleus of host cells, to regulate cell cycle control and apoptosis [13]–[16]. High-risk oncogenic HPV16 accounts for approximately 90% of HPV-associated oropharyngeal cancer [17], [18]. HPV16 may cause malignant transformation through its E6 and E7 oncoproteins [19], and inactivation of both p53 and p73 by E6 allows the cell to escape normal cell cycle checkpoints, leading to cell transformation and immortalization [19]–[21].

*p53* codon 72 polymorphism causes a change in the p53 protein sequence with a substitution of proline for arginine at codon 72, which may alter the apoptotic potential of p53 and the susceptibility of p53 to E6-mediated degradation [22]–[24], and subsequently affect the carcinogenic potential of HPV16. *p73* G4C14-to-A4T14 polymorphism at exon 2 appears to result in an alteration of gene expression possibly by altering the efficiency of translational initiation [25]. Such alteration in *p73* expression may also influence on the interaction between E6 protein and p73 and its apoptotic capacity [26]. Thus, each of these genetic variants may affect the interaction between p53 and p73 and HPV, and result in individual differences in resistance to apoptosis, which might enable HPV-infected cancer cells to escape or counterattack against the inflammation/immune responses. Therefore, such genetic variants may affect HPV clearance, subsequently contributing to tumor HPV16 status of oropharyngeal cancer patients. Since *p53* and *p73* variants can alter the affinity for or functional interactions of the E6 protein with both p53 and p73, they may jointly affect the association between tumor HPV16 status and these two polymorphisms. To test the hypothesis, the combined effect of these two putatively functional polymorphisms of *p53* and *p73* on the association was analyzed in this case-case comparison study of 309 newly diagnosed oropharyngeal cancer patients for whom tumor specimens became available.

## Materials and Methods

### Study Subjects

In this study, a total of 309 oropharyngeal cancer patients were enrolled consecutively as part of an ongoing molecular epidemiology study of SCCHN at The University of Texas M. D. Anderson Cancer Center from December 1996 to November 2009. Details for recruitment of study patients have been previously described [27]. Briefly, these patients were recruited before treatment without restrictions on age, sex, and cancer stage, and all cases were newly diagnosed and histologically confirmed oropharyngeal squamous cell carcinoma. A total of 30 ml of blood was drawn from all these patients for the genotyping. Paraffin-embedded tumor tissue samples were requested for tumor HPV16 detection. In addition, all of the patients signed informed consent and completed a questionnaire. The protocol of this study was reviewed and approved by the University of Texas M.D. Anderson Cancer Center institutional review boards.

### Tumor HPV16 Detection

The DNA from the paraffin-embedded tumor tissues of all study patients was extracted using a tissue DNA extraction kit (Qiagen Inc., Valencia, CA). Tumor tissues from the study subjects were tested for the presence of HPV16 DNA using PCR-based type-specific assays with modification and quality control for the E6 and E7 regions [28]. Assays of the samples were run in triplicate, with positive (Siha cell line) and negative (TPC-1 cell line) controls and with β-actin as DNA quality control. Each subject was classified as HPV16-positive or HPV16-negative based on tumor HPV16 DNA determination. Southern blotting analysis was performed to confirm HPV16 E6 and E7 specificity in a portion of the paraffin-embedded tissue samples, using a Roche Diagnostics labeling and hybridization system [3] (Roche Applied Science, Indianapolis, IN). HPV16 E6 and E7 specificity were also confirmed in a portion of samples by digesting the PCR products with restriction enzymes Ban II and Msp I to verify the specific fragments for E6 and E7. The results of the two methods were 100% concordant. The results of tumor HPV16 status were confirmed with 100% concordance in the repeated samples.

### *p53* and *p73* Genotyping

*p53* and *p73* polymorphisms were genotyped using genomic DNA which was isolated from patients' peripheral leukocyte pellets of blood samples. The methods for the genotyping have been previously described [29], [30]. Approximately 10% of the samples were also selected for retesting for quality control purposes, and the repeated results were 100% concordant.

### Statistical Analysis

The χ^2^ test was used to evaluate the differences in the distributions of selected demographic characteristics, tobacco smoking and alcohol drinking between HPV16^+^ and HPV16^−^ cases, and used the Student's *t* test for comparison of mean values of age between the two groups. Association of HPV16 positivity of oropharyngeal cancer patients with variant genotypes of *p53* and *p73* polymorphisms was estimated by computing the odds ratios (ORs) and 95% confidence intervals (95% CIs). Both univariate and multivariable logistic regression models were performed for the analyses. Multivariable logistic regression models were fully adjusted with age, sex, ethnicity, and smoking and alcohol status. These variables were selected for adjustment after a stepwise search strategy in developing such multivariable models. Former smokers were defined as smokers who had quit smoking at least 1 year before presentation, and former smokers were grouped with current smokers as “ever-smokers”. “Never smokers” were defined as those who had smoked fewer than 100 cigarettes in their lifetime. “Drinkers” were defined as those who had at least one alcoholic drink per week for at least 1 year, while “former drinkers” were defined as those who had quit drinking alcoholic beverages in this manner for at least 1 year before presentation. Association was considered to be statistically significant for a two-sided test set at *p*<0.05. Statistical Analysis System software (Version 9.1; SAS Institute, Cary, NC) was used for all statistical analyses.

## Results

The *p53* and *p73* genotype data, demographic characteristics, smoking status and drinking status of the patients are shown in **Table 1**. The distribution of sex and smoking status was significantly different between HPV16-positive and HPV16-negative oropharyngeal cancer patients (*P* = 0.010 for sex and *P* = 0.003 for tobacco smoking). There was no significant difference in age between tumor HPV16-positive (median, 54 years; mean, 54.0 years; and range, 28–81 years) and tumor HPV16-negative oropharyngeal cancer patients (median, 52 years; mean, 54.9 years; and range, 30–83 years). Neither was in ethnicity and alcohol drinking status between the two groups.

**Table 1:** Distribution of selected variables in patients with oropharyngeal cancer by tumor HPV16 status.

Variable	HPV16^+^ Patients (N = 230)		HPV16^−^ Patients (N = 79)		*P* value*
	No.	%	No.	%	
Age					
≤50years	77	33.5	31	39.2	0.354
>50 years	153	66.5	48	60.8	
Sex					
Male	207	90.0	62	78.5	0.010
Female	23	10.0	17	21.5	
Ethnicity					
Non-Hispanic white	216	94.0	70	88.6	0.121
Others	14	6.0	9	11.4	
Tobacco smoking					
Ever	119	51.7	56	70.9	0.003
Never	111	48.3	23	29.1	
Alcohol drinking					
Ever	179	77.8	61	77.2	0.910
Never	51	22.2	18	22.8	

* Two-sided χ^2^ test.

The results of genotype distributions and allele frequencies of *p73* and *p53* in HPV16-positive and HPV16-negative patients are summarized in **Table 2**. The AT and Pro variant alleles of *p73* and *p53* were significantly more common among HPV16-positive patients (26.1% for *p73* and 23.7% for *p53*) than among HPV16-negative patients (16.5% for *p73* and 15.2% for *p53*) (*P* = 0.020 for *p73* and *P* = 0.043 for *p53*), indicating that the AT and Pro alleles may be associated with tumor HPV16-positivity among oropharyngeal cancer patients. Compared with the wild-type GC/GC homozygote, the combined GC/AT+AT/AT variant genotypes were associated significantly with tumor HPV16-positive oropharyngeal cancer (OR, 2.1, 95% CI, 1.2–3.8). Furthermore, the dose-effect relationship between the number of the AT alleles and the tumor HPV16-positive oropharyngeal cancer was statistically significant (*P* = 0.010). For *p53* polymorphism, both Arg/Pro and Pro/Pro genotypes were found to have no association with HPV16-positive oropharyngeal tumors (OR 1.2, 95% CI 0.6–2.5; and OR 1.1, 95% CI 0.1–9.1, respectively). whereas compared with the *p53* Arg/Arg homozygote, the combined Arg/Pro+Pro/Pro variant genotypes were significantly associated with tumor HPV16-positive oropharyngeal cancer (OR, 1.9, 95% CI, 1.1–3.3).

**Table 2:** Association of tumor HPV16 positivity of patients with oropharyngeal cancer with *p73* and *p53* Genotypes.

	HPV16^+^ Patients (N = 230)		HPV16^−^ Patients (N = 79)		Crude OR	Adjusted OR
Genotypes	No.	%	No.	%	(95% CI)	(95% CI)^a^
*p73* G4C14-to-A4T14						
GC/GC^b^	123	53.5	55	69.6	1.0	1.0
GC/AT	94	40.9	22	27.9	1.9 (1.1–3.4)	2.1 (1.1–3.7)
AT/AT	13	5.6	2	2.5	2.9 (0.6–13.3)	3.0 (0.6–14.5)
Combined variant genotypes						
GC/AT+AT/AT	107	46.5	24	30.4	2.0 (1.2–3.4)	2.1 (1.2–3.8)
*p53* Arg/Pro						
Arg/Arg^b^	130	56.5	56	70.9	1.0	1.0
Arg/Pro	91	39.6	22	27.8	1.3 (0.6–2.7)	1.2 (0.6–2.5)
Pro/Pro	9	3.9	1	1.3	1.1 (0.1–10.5)	1.1 (0.1–9.1)
Combined variant genotypes						
Arg/Pro+Pro/Pro	100	43.5	23	29.1	1.9 (1.1–3.2)	1.9 (1.1–3.3)

a Adjusted for age, sex, ethnicity, smoking and alcohol use in a logistic regression model.

b Reference group.

No interaction effect between these two polymorphisms on tumor HPV16 status in oropharyngeal cancer patients was observed (*P_int_*_._ = 0.374), while the oropharyngeal cancer patients with variant genotypes of both *p53* and *p73* polymorphisms were more likely to have HPV16-positive tumors. Therefore, to evaluate the association of tumor HPV16 status with combined risk genotypes of both polymorphisms, the study subjects were categorized into three main groups based on the level of association of tumor HPV16 positivity with variant genotypes of each polymorphism (**Table 3**): 1) the low-risk group (if subjects with *p53* Arg/Arg and *p73* GC/GC genotypes); 2) the medium-risk group (if subjects with *p53* Arg/Arg and *p73* AT carriers or *p53* Pro carriers and *p73* GC/GC genotypes); and 3) the high-risk group (if subjects with *p53* Pro carriers and *p73* AT carriers), respectively. Compared with the low-risk group, both the medium-risk and high-risk groups exhibited a significant association with tumor HPV16 positivity (OR, 2.4, 95% CI, 1.3–4.2 and OR, 3.2, 95% CI, 1.4–7.4, respectively). The dose-effect relationship between the combined *p53* and *p73* variant genotypes and tumor HPV16 positivity in oropharyngeal cancer was also statistically significant (*p* = 0.001).

**Table 3:** Association of tumor HPV16 positivity of patients with oropharyngeal cancer with combined *p73* and *p53* variant genotypes.

Combined *p53* and *p73* variant genotypes^a^	HPV16^+^ Patients (N = 230)		HPV16^−^ Patients (N = 79)		Crude OR (95% CI)	Adj. OR (95% CI)^b^
	No.	%	No.	%		
Low-risk group	72	31.3	41	51.9	1.0 (ref.^c^)	1.0 (ref.^c^)
Medium-risk group	109	47.4	29	36.7	2.1 (1.2–3.8)	2.4 (1.3–4.2)
High-risk group	49	21.3	9	11.4	3.1 (1.4–7.0)	3.2 (1.4–7.4)
Trend test					P = 0.001	*P* = 0.001

a Low-risk group: individuals with *p53* Arg/Arg and *p73* GC/GC genotypes; Medium-risk group: individuals with *p53* Arg/Arg and *p73* AT carriers or *p53* Pro carriers and *p73* C/GC; and High-risk group: individuals with *p53* Pro carriers and *p73* AT carriers.

b ORs were adjusted for age, sex, ethnicity, smoking, and alcohol use in a logistic regression model.

c Reference group.

The stratified analyses by age, sex, ethnicity, smoking status, and alcohol status are shown in **Table 4**, and the association was further evaluated with adjustment for the aforementioned variables. The association was more pronounced among patients who were older, men, non-Hispanic white, never-smokers, and ever drinkers. For example, compared with the low-risk group, the high-risk group exhibited a greater association with HPV16-positive tumor status among male patients (OR, 3.5, 95% CI, 1.4–8.6) and in never smokers (adjusted OR, 5.0, 95% CI, 1.0–24.7) as opposed to a non-significant association among female patients (OR, 2.1, 95% CI, 0.1–30.0) and an OR of 3.0 in ever smokers (OR, 3.0, 95% CI, 1.0–8.2). Furthermore, a significant dose-effect relationship between combined *p53* and *p73* variant genotypes and tumor HPV16 positivity in oropharyngeal cancer was also observed among several subgroups, such as in patients who were older, men, non-Hispanic white, and never smokers (*p*<0.01).

**Table 4:** Stratified analysis of associations between combined *p73* and *p53* variant genotypes and tumor HPV16 status among oropharyngeal cancer patients.

Variables	Adjusted OR (95% CI)						
	Low-Risk Group^a^	OR^b^	Medium-Risk Group^a^	OR^b^, 95% CI	High-Risk Group^a^	OR^b^, 95% CI	Trend Test
	CASE/CNTL^c^		CASE/CNTL^c^		CASE/CNTL^c^		
Total	72/41	1.0^d^	109/29	2.4 (1.3–4.2)	49/9	3.2 (1.4–7.4)	<0.01
Age (years)							
≤50	27/12	1.0	32/14	1.3 (0.4–3.6)	18/5	2.1 (0.6–7.5)	0.285
>50	45/29	1.0	77/15	3.9 (1.8–8.3)	31/4	5.0 (1.5–16.0)	<0.01
Sex							
Male	64/33	1.0	97/21	2.8 (1.4–5.4)	46/8	3.5 (1.4–8.6)	0.001
Female	8/8	1.0	12/8	1.4 (0.3–5.8)	3/1	2.1 (0.1–30.0)	0.546
Ethnicity							
Non-Hispanic White	66/36	1.0	102/26	2.4 (1.3–4.5)	48/8	3.6 (1.5–8.7)	<0.01
Others	6/5	1.0	7/3	8.8 (0.5–146.0)	1/1	5.8 (0.1–291.2)	0.225
Smoking							
Never	38/15	1.0	51/6	3.9 (1.3–11.5)	22/2	5.0 (1.0–24.7)	0.011
Ever	34/26	1.0	58/23	2.0 (0.9–4.1)	27/7	3.0 (1.0–8.2)	0.020
Alcohol							
Never	17/9	1.0	23/8	2.4 (0.6–8.7)	11/1	6.1 (0.6–62.7)	0.080
Ever	55/32	1.0	86/21	2.6 (1.3–5.1)	38/8	2.8 (1.1–6.9)	<0.01

a Low-Risk group: individuals with *p53* Arg/Arg and *p73* GC/GC genotypes; Medium-Risk group: individuals with *p53* Arg/Arg and *p73* AT carriers or *p53* Pro carriers and *p73* GC/GC; and High-Risk group: individuals with *p53* Pro carriers and *p73* AT carriers.

b ORs were adjusted for age, sex, ethnicity, smoking status, and alcohol use in a logistic regression model.

c CASE/CNTL: HPV16^+^/HPV16^−^ patients.

d Low-risk group was used as the reference group.

## Discussion

We and others have previously assessed associations of these two polymorphisms with HPV-associated SCCHN or their subgroups in several studies [31]–[35], while these studies categorized HPV16 status of study patients based on serology or included mixed cancer sites due to the unavailability of tumor status in our previous studies [31], [32], [34], [35]. These studies suggest that HPV tumor positivity may have powerful prognostic effect on outcomes of oropharyngeal cancer, whereas these results are not in agreement with the findings of others [36]–[38]. It should be noted that other prognostic variables, including patient demographics, tumor site and stage, and treatment may also significantly affect the outcomes of oropharyngeal cancer. Particularly, a later stage, nodal involvement and advanced grade were frequently seen in HPV-positive cancers [12]. Therefore, to guide treatment recommendations for the future, the suggestion that HPV tumor positivity is a favorable prognostic marker needs to be viewed critically given that significant confounding is not controlled for a variety of independent prognostic variables.

The data from this study suggest that variant genotypes of each polymorphism may individually, and more likely jointly, influence on tumor HPV16 status in oropharyngeal cancer and could be potentially susceptible markers for the tumor HPV16-positive patients. This study with tumor-based HPV16 status and a homogenous subgroup of SCCHN patients would help more accurately evaluate the associations between the *p53* and *p73* polymorphisms and tumor HPV16-positive oropharyngeal cancers. Although the precise mechanism by which these polymorphisms affect the tumor HPV16 status of oropharyngeal cancer has not yet been clarified, there are some biologically plausible explanations. Firstly, p53 and p73 proteins structurally have similar domain structures and very high amino acid identities in DNA-binding domain [39]. Functionally, these two proteins have some common target genes, and may play similar roles in regulation of several cellular activities such as cell cycle control, DNA repair, and apoptosis [13]–[16]. Additionally, both p53 and p73 can interact with HPV16 by being directly bound to and subsequently degraded or inactivated by oncoprotein E6 [20], [21], [40], and p73 may compensate for the loss of p53 function in some human malignancies. Furthermore, p73 can promote apoptosis via the E2F-p73 pathway and inactivation of p73 by oncogenic HPV16 E6 appears to be analogous to its inactivation of p53 without the modulation of the DNA-binding activities [28], [41]. Finally, unlike p53, p73 is resistant to degradation by HPV16 E6, can suppress cell growth, and induce apoptosis in HPV16 E6-expressing cells [42]. It is our speculation that *p53* and *p73* polymorphisms may be jointly associated with tumor HPV16 status in oropharyngeal cancer through interaction among HPV16 oncoprotein E6, p53 and p73.

Several studies have reported that *p53* codon 72 and *p73* G4C14-to-A4T14 polymorphisms were significantly associated with risk of HPV16-associated squamous cell carcinoma of the oropharynx [31]–[33]. Perrone, et al. found that *p53* 72RP genotype may have a protective effect on risk of oropharyngeal cancer, while the PP genotype is associated with HPV16-positive tumors [33]. The discrepancy between these findings and our current findings might be, at least in part, explained by following several factors including differences in race, small sample sizes, differences in study designs, and lack of detailed information on smoking and alcohol use.

Stratified analyses have shown that association between combined *p53* and *p73* variant genotypes and tumor HPV16 positivity in oropharyngeal cancer was more pronounced among never-smoker patients. This result may provide additional support for findings in several previous studies, in which it was reported that a significant proportion of oropharyngeal cancers were driven by HPV, while most nonoropharyngeal cancers were caused by smoking and drinking [43], [44]. As HPVs have evolved several mechanisms to bypass immune recognition or killing, *p53* and *p73* polymorphisms possibly modulate the apoptotic capacity of the host to clear cells infected with HPV through inflammation/immune systems, which control the HPV clearance and escape of immune surveillance, subsequently affecting the tumor HPV status [45]. However, these hypotheses need to be tested in future studies.

The oropharyngeal cancer patients who were moderate to heavy drinkers were less likely to be tumor HPV-positive [3], whereas association between tumor HPV16 positivity and combined *p53* and *p73* variant genotypes in oropharyngeal cancer were more evident in ever-drinkers and men in current study (adjusted OR, 2.8, 95% CI, 1.1–6.9 for ever drinkers and OR, 3.5, 95% CI, 1.4–8.6 for male patients), suggesting HPV16 infection may act synergistically with alcohol and/or tobacco exposure, although nonsmokers/nondrinkers were more likely to have HPV-positive oropharyngeal cancer than smokers/drinkers [46]. In addition, ethanol consumption may synergize with *p53* and *p73* variants to increase susceptibility to HPV16 infection through either suppression of immune responses or changes in sexual behaviors. However, further analyses could not be performed qualitatively as data on specificity, intensity and duration of alcohol exposure were limited in this study. When compared with the finding reported in another study [3], we found that the association between HPV16 positivity and combined *p53* and *p73* risk genotypes was of significance in old patients. A simple explanation for the inconsistent findings follows. Young patients may have strong immune response generated against an HPV infection compared with old patients and thus have strong ability of the host to clear cells infected with HPV, less likely having HPV16-positive tumors. However, all these hypotheses mentioned above need to be tested in future large studies.

Strengths of this study include analysis of single tumor site (only oropharyngeal cancer patients), HPV16 tumor status instead of serology, and careful quality control in genotyping. Our analysis among only oropharyngeal cancer patients minimizes the issue of the confounding effect from mixed tumor sites, and determination of HPV16 tumor status instead of serology greatly improves classification of study patients and accuracy of the association in this analysis. Although our study has such several strengths, interpretation of our findings may be limited for several main reasons. First, compared with HPV16-negative cancer patients, HPV16-positive cancer patients have distinct clinical characteristics, demographic variables and epidemiological risk factors. Thus, it is difficult to match these factors in such a study. However, in current analysis, our study was adjusted for age, sex, ethnicity, tobacco smoking and alcohol drinking, and the potential effect of confounding factors on this association should be minimized. Second, the sample sizes in each stratum of the analyses were relatively small, and our estimates of association could be observed by chance. Third, misclassification of tumor HPV16 status could occur due to the presence of lower copies of HPV in some tumor cells [47]. Finally, our study was not population-based case-control study design instead of a case-case comparison. We did not measure exposure to HPV16, and thus the control group of tumor HPV16-negative patients may not adequately represent the true prevalence of HPV 16 exposure in the general population.

In conclusion, our study demonstrated that the combined variant genotypes of *p53* codon 72 and *p73* G4C14-to-A4T14 polymorphisms individually, and more likely jointly, had a significantly effect on tumor HPV16 status in patients with oropharyngeal cancer, particularly in never-smoker patients. However, further studies with larger sample sizes are needed to verify our findings.

## References

[1] Vokes EE, Weichselbaum RR, Lippman SM, Hong WK (1933). Head and neck cancer.. N Engl J Med.

[2] Dobrossy L (2005). Epidemiology of head and neck cancer: magnitude of the problem.. Cancer Metastasis Rev.

[3] Gillison ML, Koch WM, Capone RB, Spafford M, Westra WH (2000). Evidence for a causal association between human papillomavirus and a subset of head and neck cancers.. J Natl Cancer Inst.

[4] Koch WM, Lango M, Sewell D, Zahurak M, Sidransky D (1999). Head and neck cancer in nonsmokers: a distinct clinical and molecular entity.. Laryngoscope.

[5] Haughey BH, Hinni ML, Salassa JR, Hayden RE, Grant DG (2011). Transoral laser microsurgery as primary treatment for advanced-stage oropharyngeal cancer: a United States multicenter study.. Head Neck.

[6] Rich JT, Liu J, Haughey BH (2011). Swallowing function after transoral laser microsurgery (TLM) ± adjuvant therapy for advanced-stage oropharyngeal cancer.. Laryngoscope.

[7] Gillison ML, D'Souza G, Westra W, Sugar E, Xiao W (2008). Distinct risk factor profiles for human papillomavirus type 16-positive and human papillomavirus type 16-negative head and neck cancers.. J Natl Cancer Inst.

[8] Ritchie JM, Smith EM, Summersgill KF, Hoffman HT, Wang D (2003). Human papillomavirus infection as a prognostic factor in carcinomas of the oral cavity and oropharynx.. Int J Cancer.

[9] Mellin H, Friesland S, Lewensohn R, Dalianis T, Munck-Wikland E (2002). Human papillomavirus (HPV) DNA in tonsillar cancer: clinical correlates, risk of relapse, and survival.. Int J Cancer.

[10] Li W, Thompson CH, O'Brien CJ, McNeil EB, Scolyer RA (2003). Human papillomavirus positivity predicts favourable outcome for squamous carcinoma of the tonsil.. Int J Cancer.

[11] Lindel K, Beer KT, Laissue J, Greiner RH, Aebersold DM (2001). Human papillomavirus positive squamous cell carcinoma of the oropharynx: a radiosensitive subgroup of head and neck carcinoma.. Cancer.

[12] Smith EM, Ritchie JM, Summersgill KF, Klussmann JP, Lee JH (2004). Age, sexual behavior and human papillomavirus infection in oral cavity and oropharyngeal cancers.. Int J Cancer.

[13] Vogelstein B, Lane D, Levine AJ (2000). Surfing the p53 network.. Nature.

[14] Helton ES, Chen X (2000). p53 modulation of the DNA damage response.. J Cell Biochem.

[15] Jost CA, Marin MC, Kaelin WG (1997). p73 is a simian [correction of human] p53-related protein that can induce apoptosis.. Nature.

[16] Melino G, De Laurenzi V, Vousden KH (2002). p73: friend or foe in tumorigenesis.. Nat Rev Cancer.

[17] Fakhry C, Gillison ML (2006). Clinical implications of human papillomavirus in head and neck cancers.. J Clin Oncol.

[18] Herrero R, Castellsague X, Pawlita M, Lissowska J, Kee F (2003). Human papillomavirus and oral cancer: the International Agency for Research on Cancer multicenter study.. J Natl Cancer Inst.

[19] Munger K, Howley PM (2002). Human papillomavirus immortalization and transformation functions.. Virus Res.

[20] Scheffner M, Werness BA, Huibregtse JM, Levine AJ, Howley PM (1990). The E6 oncoprotein encoded by human papillomavirus types 16 and 18 promotes the degradation of p53.. Cell.

[21] Scheffner M, Huibregtse JM, Vierstra RD, Howley PM (1993). The HPV-16 E6 and E6-AP complex functions as a ubiquitin-protein ligase in the ubiquitination of p53.. Cell.

[22] Thomas M, Kalita A, Labrecque S, Pim D, Banks L (1999). Two polymorphic variants of wild-type p53 differ biochemically and biologically.. Mol Cell Biol.

[23] Dumont P, Leu JI, Della Pietra AC, George DL, Murphy M (2003). The codon 72 polymorphic variants of p53 have markedly different apoptotic potential.. Nat Genet.

[24] Storey A, Thomas M, Kalita A, Harwood C, Gardiol D (1998). Role of a p53 polymorphism in the development of human papillomavirus-associated cancer.. Nature.

[25] Kaghad M, Bonnet H, Yang A, Creancier L, Biscan JC (1997). Monoallelically expressed gene related to p53 at 1p36, a region frequently deleted in neuroblastoma and other human cancers.. Cell.

[26] Haber DA, Fearon ER (1998). The promise of cancer genetics.. Lancet.

[27] Guan X, Sturgis EM, Lei D, Liu Z, Dahlstrom KR (2010). Association of TGF-β1 Genetic Variants with HPV16-positive Oropharyngeal Cancer.. Clin Cancer Res.

[28] Park JS, Kim EJ, Lee JY, Sin HS, Namkoong SE (2001). Functional inactivation of p73, a homolog of p53 tumor suppressor protein, by human papillomavirus E6 proteins.. Int J Cancer.

[29] Li G, Sturgis EM, Wang LE, Chamberlain RM, Amos CI (2004). Association of a p73 exon 2 G4C14-to-A4T14 polymorphism with risk of squamous cell carcinoma of the head and neck.. Carcinogenesis.

[30] Li F, Sturgis EM, Zafereo M, Wei Q, Li G (2010). Association of p53 codon 72 polymorphism with risk of second primary malignancy in patients with squamous cell carcinoma of the head and neck.. Cancer.

[31] Ji X, Neumann AS, Sturgis EM, Wei Q, Li G (2008). p53 codon 72 polymorphism associated with risk of human papillomavirus-associated squamous cell carcinoma of the oropharynx in never smokers.. Carcinogenesis.

[32] Chen X, Sturgis EM, Etzel CJ, Wei Q, Li G (2008). *p73* G4C14-to-A4T14 polymorphism and risk of human papillomavirus associated squamous cell carcinoma of the oropharynx in never smokers and never drinkers.. Cancer.

[33] Perrone F, Mariani L, Pastore E, Orsenigo M, Suardi S (2007). p53 codon 72 polymorphisms in human papillomavirus-negative and human papillomavirus-positive squamous cell carcinomas of the oropharynx.. Cancer.

[34] Chen X, Sturgis EM, El-Naggar AK, Wei Q, Li G (2008). Combined effects of the p53 codon 72 and p73 G4C14-to-A4T14 polymorphisms on the risk of HPV16-associated oral cancer in never-smokers.. Carcinogenesis.

[35] Ji X, Sturgis EM, Zhao C, Wei Q, Li G (2009). Association of p73 G4C14-to-A4T14 polymorphism with human papillomavirus type 16 status in squamous cell carcinoma of the head and neck in non-Hispanic Whites.. Cancer.

[36] Pintos J, Franco EL, Black MJ, Bergeron J, Arella M (1999). Human papillomavirus and prognoses of patients with cancers of the upper aerodigestive tract.. Cancer.

[37] Báez A, Almodóvar JI, Cantor A, Celestin F, Cruz-Cruz L (2004). High frequency of HPV16-associated head and neck squamous cell carcinoma in the Puerto Rican population.. Head Neck.

[38] Koskinen WJ, Chen RW, Leivo I, Mäkitie A, Bäck L (2003). Prevalence and physical status of human papillomavirus in squamous cell carcinomas of the head and neck.. Int J Cancer.

[39] Melino G, Lu X, Gasco M, Crook T, Knight RA (2003). Functional regulation of p73 and p63: development and cancer.. Trends Biochem Sci.

[40] Werness BA, Levine AJ, Howley PM (1990). Association of human papillomavirus types 16 and 18 E6 proteins with p53.. Science.

[41] Phillips AC, Vousden KH (2001). E2F-1 induced apoptosis.. Apoptosis.

[42] Das S, Somasundaram K (2006). Therapeutic potential of an adenovirus expressing p73 beta, a p53 homologue, against human papillomavirus positive cervical cancer in vitro and in vivo.. Cancer Biol Ther.

[43] Dahlstrom KR, Adler-Storthz K, Etzel CJ, Wei Q, Strugis EM (2003). Human papillomavirus type 16 infection and squamous cell carcinoma of the head and neck in never-smokers: a matched pair analysis.. Clin Cancer Res.

[44] Hammarstedt L, Lindquist D, Dahlstrand H, Romanitan M, Dahlgren LO (2006). Human papillomavirus as a risk factor for the increase in incidence of tonsillar cancer.. Int J Cancer.

[45] Favre A, Paoli D, Poletti M, Marzoli A, Giampalmo A (1986). The human palatine tonsil studied from surgical specimens at all ages and in various pathological conditions. 1. Morphological and structural analyses.. Z Mikrosk Anat Forsch.

[46] Fouret P, Monceaux G, Temam S, Lacourreye L, St Guily JL (1997). Human papillomavirus in head and neck squamous cell carcinomas in nonsmokers.. Arch Otolaryngol Head Neck Surg.

[47] Huang CC, Qiu JT, Kashima ML, Karman RJ, Wu TC (1998). Generation of type-specific probes for the detection of single-copy human papillomavirus by a novel in situ hybridization method.. Mod Pathol.

